# Macrophage Polarization Mediated by Mitochondrial Dysfunction Induces Adipose Tissue Inflammation in Obesity

**DOI:** 10.3390/ijms23169252

**Published:** 2022-08-17

**Authors:** Long Xu, Xiaoyu Yan, Yuanxin Zhao, Jian Wang, Buhan Liu, Sihang Yu, Jiaying Fu, Yanan Liu, Jing Su

**Affiliations:** Key Laboratory of Pathobiology, Department of Pathophysiology, Ministry of Education, College of Basic Medical Sciences, Jilin University, 126 Xinmin Street, Changchun 130021, China

**Keywords:** obesity, adipose tissue inflammation, mitochondrial dysfunction, NLRP3 inflammasome, insulin resistance

## Abstract

Obesity is one of the prominent global health issues, contributing to the growing prevalence of insulin resistance and type 2 diabetes. Chronic inflammation in adipose tissue is considered as a key risk factor for the development of insulin resistance and type 2 diabetes in obese individuals. Macrophages are the most abundant immune cells in adipose tissue and play an important role in adipose tissue inflammation. Mitochondria are critical for regulating macrophage polarization, differentiation, and survival. Changes to mitochondrial metabolism and physiology induced by extracellular signals may underlie the corresponding state of macrophage activation. Macrophage mitochondrial dysfunction is a key mediator of obesity-induced macrophage inflammatory response and subsequent systemic insulin resistance. Mitochondrial dysfunction drives the activation of the NLRP3 inflammasome, which induces the release of IL-1β. IL-1β leads to decreased insulin sensitivity of insulin target cells via paracrine signaling or infiltration into the systemic circulation. In this review, we discuss the new findings on how obesity induces macrophage mitochondrial dysfunction and how mitochondrial dysfunction induces NLRP3 inflammasome activation. We also summarize therapeutic approaches targeting mitochondria for the treatment of diabetes.

## 1. Introduction

Metabolic syndrome includes a range of diseases associated with insufficient physical activity and overnutrition [1]. Studies over the past two decades have shown that chronic inflammation is the key driver of these diseases [2,3,4,5]. Type 2 diabetes (T2D) is a common and serious complication of metabolic syndrome. Although T2D can exist in isolation, many patients with T2D meet the diagnostic criteria of metabolic syndrome [6]. Insulin resistance and β-cell dysfunction are two major components of T2D pathology. Most people with type 2 diabetes are overweight or obese. Chronic inflammation in adipose tissue is considered to be a key risk factor for the development of insulin resistance and T2D in obese individuals. Human and animal studies conducted in the 1990s provide the initial mechanistic evidence supporting the inflammatory origin of obesity and diabetes. In these studies, increased inflammatory modifications were observed in adipose tissue from obese rodents and humans, and enhanced secretion of pro-inflammatory cytokine tumor necrosis factor-α(TNF-α) was able to induce insulin resistance by inactivating insulin receptor substrate 1 (IRS-1) [2,7].

Macrophages play a key role in chronic adipose tissue inflammation. In lean mice and humans, macrophages comprise about 5% of total cells in adipose tissue, during obesity they comprise 50% of all adipose tissue cells [8]. Besides the increase in macrophage numbers, the localization and phenotype of adipose tissue macrophages (ATMs) are altered during obesity. In the lean state, ATMs are distributed throughout adipose tissue and display limited inflammatory properties. In obese adipose tissue, ATMs are located around dead adipocytes, form so-called crown-like structures (CLSs) and display proinflammatory features [9,10,11]. The presence of macrophages in CLSs within obese adipose tissue was directly associated with insulin resistance [12,13].

The unique ability of macrophages to rapidly adapt to changes in microenvironment cause them to embrace a variety of phenotypes ranging from anti-inflammatory to pro-inflammatory. In almost every tissue, macrophages play a key role in maintaining tissue homeostasis by removing cellular debris, participating in tissue immune surveillance and diminishing inflammation [14]. It is a simplified operational framework to use the concept of M1-like and M2-like polarization of macrophages to describe an essentially heterogeneous population of tissue macrophages. In lean adipose tissue, macrophages exhibit an anti-inflammatory M2-like phenotype and maintain the homeostasis of adipose tissue [15]. However, in obesity adipose tissue, ATM polarity shifts towards the proinflammatory M1-like phenotype, and this shift leads to the secretion of pro-inflammatory cytokines, which can impair the insulin signal transduction and promote insulin resistance.

M1 and M2 macrophages have different metabolic features. M1 macrophages obtain energy mainly by glycolysis, while M2 macrophages utilize oxidative metabolism. Recent studies have shown that shifts in mitochondrial metabolism and physiology play an important role in the recruitment of macrophages to different activation states, such as alterations in oxidative metabolism, mitochondrial reactive oxygen species (mtROS), mitochondrial ultrastructure, and membrane potential [16]. Obesity-induced expansion of adipose tissue results in local tissue hypoxia and release of fatty acids (FAs), which can induce mitochondrial dysfunction in ATMs. Mitochondrial quality control (MQC) is essential to maintain the normal structure and physiological function of mitochondria, and impaired MQC will lead to the accumulation of dysfunctional mitochondria. Mitochondrial dysfunction leads to altered cellular metabolism, which may lead to abnormal activation of macrophages and the development of certain diseases. Accumulation of ROS and loss of membrane potential in dysfunctional mitochondria lead to disrupted mitochondrial bilayer membrane structure and the release of mitochondrial damage-associated molecular patterns (DAMPs), which ultimately leads to the activation of inflammasomes and the release of inflammatory cytokines.

NLR family pyrin domain containing 3 (NLRP3) is the most studied inflammasome protein in metabolic studies and plays a crucial role in immune response, glucose homeostasis, lipid metabolism, and the function of adipocytes [17]. Mitochondrial dysfunction and subsequent accumulation of mtROS, mitochondrial DNA (mtDNA) and mitochondria-associated proteins and lipids play a crucial role in the activation of the NLRP3 inflammasome [18]. Activation of the NLRP3 inflammasome promotes the release of IL-1β, which not only impairs peripheral insulin sensitivity but also interferes with adipose tissue endocrine and immune function in a paracrine manner [17]. Inhibition of the NLRP3 inflammasome reduces IL-1β release and adipose tissue inflammation and ultimately slows down progression of T2D [19].

In this review, we discuss the role of ATM in low-grade adipose tissue inflammation and insulin resistance, focusing on the effect of mitochondrial dysfunction on macrophage polarization and the contribution of macrophages inflammatory responses to insulin resistance. Mitochondrial dysfunction leads to the activation of the NLRP3 inflammasome and the release of inflammatory cytokines. Increased understanding of mitochondrial dysfunction-mediated macrophage polarization may provide new insights into the treatment of diabetes.

## 2. Macrophage Polarization and Cellular Energy Metabolism

Macrophages are an important type of immune cell that have attracted much attention as important contributors to adipose tissue function. Compared with lean adipose tissue, the number of macrophages in obese adipose tissue was significantly increased. Besides increasing in number, ATMs also change their phenotype during obesity. The majority of the macrophage population in the adipose tissue of obese mice consists of “classically activated” M1 macrophages, characterized by increased secretion of inflammatory cytokines [11]. In contrast, the majority of macrophages in lean adipose tissue were identified as “alternately activated” M2 macrophages [11,15]. In addition to their well-characterized gene and protein expression, significant differences between M1 and M2 macrophages in terms of cellular metabolism have been identified. The cellular metabolism of macrophages determines their polarization phenotype.

### 2.1. M1 Macrophages

In the resting state, macrophages obtain energy mainly by oxidative phosphorylation (OXPHOS). After lipopolysaccharide (LPS) stimulation, macrophages polarize toward an M1 phenotype. Uptake of glucose increases, and aerobic glycolysis becomes the main metabolic pathway [20]. Increased glucose uptake increases glycolytic flux and the expression of pyruvate kinase muscle isozyme M2 (PKM2), facilitating pyruvate production to support lactate production and supply the tricarboxylic acid cycle (TCA cycle) [21]. This altered metabolism, known as the Warburg effect, results in the accumulation of ATP [22]. The Warburg effect is primarily associated with cells undergoing rapid proliferation, but in M1 macrophages, increased glucose consumption is associated with rapid cytokine production and enhanced antibacterial activity through ROS generation [23]. Although glycolysis is a comparatively inefficient means to produce ATP, it is sufficient for macrophage function when there is adequate glucose available. Glycolytic intermediates not only can be used for the synthesis of nucleotides and amino acids, but also provide substrates for the synthesis of NADPH through the pentose phosphate pathway [24]. M1 macrophages express inducible nitric oxide synthase (iNOS), which can metabolize L-arginine to generate nitric oxide (NO) and L-citrulline [20]. NO and NO-derived reactive nitrogen species can inactivate the mitochondrial electron transport chain (ETC) [25]. Decreased activity of the ETC leads to the production of mtROS and cellular oxidative stress. Besides enhanced glycolysis, a combined metabolomics and transcriptomics approach revealed that M1 macrophages have a “broken” TCA cycle [26]. A “break” is the decrease in expression of isocitrate dehydrogenase (IDH), leading to the decreased production of α-ketoglutarate and the accumulation of citrate, which can then support FA synthesis [27]. Another “break” occurs after succinate, and there is a new pathway known as the aspartate succinate shunt, which can generate arginine to support NO production [16]. The decreased activity of succinate dehydrogenase (SDH) inhibits the conversion of succinate to fumarate, resulting in the accumulation of succinate in M1 macrophages. Succinate is a pro-inflammatory metabolite that not only promotes succinate dehydrogenase activity and mtROS production, but also enhances hypoxia-inducible factor-1α (HIF-1α) stability and subsequent IL-1β expression [21].

### 2.2. M2 Macrophages

In contrast to inflammatory macrophages, IL-4-induced macrophages exhibit enhanced mitochondrial oxidative metabolism. In addition to glucose, M2 macrophages also metabolize FA. The TCA cycle in M2 macrophages is intact and coupled to OXPHOS [26]. After IL-4 stimulation, FA uptake and fatty acid oxidation (FAO) in M2 macrophages were significantly increased to supply mitochondrial OXPHOS. Another difference between M2 macrophages and M1 macrophages is that M2 macrophages express arginase, which breaks down arginine into ornithine and urea. Ornithine can further participate in polyamine and proline synthesis, which is important for cell proliferation and tissue repair. Inhibition of FAO and/or OXPHOS both decrease arginase activity in macrophages and inhibit macrophage M2 polarization [28,29]. The activation of FAO and mitochondrial biogenesis-related pathways induced by peroxisome proliferator-activated receptor (PPAR)-γ and PPAR-γ coactivator-1β (PGC-1β) underpins the altered metabolic status that occurs with M2 differentiation [29]. Lysosomal lipolysis is the major source of FAs required by FAO in M2 macrophages [30]. Inhibition of lipolysis in M2 macrophages by orlistat inhibits OXPHOS but has no effect on the metabolism of M1 macrophages [28]. Orlistat treatment also decreased the expression of M2 macrophage markers, indicating the important role of FAO in M2 macrophages [28]. Although several studies have shown that the M2 phenotype requires FAO, the link may be less straightforward. Specific knockdown of carnitine palmitoyl transferase (CPT) 2 in macrophages reveals that bone marrow-derived macrophages (BMDM) can still express the M2 marker in the absence of FAO [31]. Whether FAO is required for M2 macrophage function or not, it is clear that FAO is associated with this anti-inflammatory cell type [32].

## 3. Mitochondrial Dysfunction of Macrophages in Obese Adipose Tissue

Activated macrophages in adipose tissue play an important role in the progression of obesity. Mitochondria are critical for the regulation of macrophage activation, polarization, and survival. In response to various extracellular signals, changes in mitochondrial metabolism and physiology may underlie the corresponding state of macrophage activation. During obesity, the progressive accumulation of lipids in adipocytes leads to hypertrophy and hyperplasia of adipose tissue, resulting in the infiltration of macrophages in adipose tissue. In adipose tissue, macrophages buffer lipolysis by absorbing and storing lipids released from adipocytes, ensuring that lipids are gradually released into the blood [33]. In addition, ATMs are responsible for removing dead or hypertrophied adipocytes from adipose tissue. These processes may lead to the accumulation of lipids within macrophages. Excessive lipid accumulation may lead to mitochondrial dysfunction, exhibiting defective β-oxidation and increased oxidative stress [34]. The increase in lipid accumulation in macrophages resembles the atherosclerosis-promoting process, in which lipid-laden macrophages accumulate and form a foamy-appearing cytoplasm [35], which inspires foam cells in atherosclerosis. Lipid overload causes endoplasmic reticulum (ER) stress [36], autophagy defects [37], lysosomal dysfunction, and mitochondrial dysfunction [38,39] in macrophages.

Hypertrophy and hyperplasia of adipocytes also lead to local tissue hypoxia. Hypoxia induces alterations in mitochondrial structure, dynamics, and genomic stability, resulting in decreased mitochondrial respiration, overproduction of ROS, loss of ATP, increased oxidative damage, and accumulation of mtDNA mutations [40,41]. Several studies have shown that hypoxic stress is significantly increased in the adipose tissue in obese mice and humans [42,43,44]. Under hypoxia, activated HIF-1α shifts cellular energy metabolism from OXPHOS to glycolysis and promotes M1 macrophage polarization and the recruitment of monocytes [45,46,47]. HIF-1α deletion in monocytes/macrophages reduces adipose tissue inflammation and improves insulin resistance in obese mice [48].

During obesity, hypertrophic adipose tissue also releases excess free fatty acids (FFAs). In a variety of cells, including macrophages, saturated fatty acids induce disruption of ER structure and function and induce unfolded protein responses (UPR) [49,50,51]. ER stress induces mitochondrial calcium overload and mitochondrial dysfunction [52]. Excessive uptake of FFAs by ATMs shifts FA utilization from OXPHOS to triglyceride, phospholipid, and ceramide synthesis, promoting lipotoxicity and the M1 phenotype [53,54]. Lipotoxicity may underlie mitochondrial dysfunction. Mitochondria are the main production site of ROS in cells and the FAs accumulated around mitochondria are peroxidized by ROS. These lipid peroxides may induce mitochondrial DNA, RNA, and protein damage, leading to mitochondrial dysfunction. FAs, a kind of mtDAMP, can induce the inflammatory responses of macrophages, leading to obesity-related insulin resistance. Saturated fatty acids induce the activation of the NLRP3 inflammasome in macrophages by promoting mtROS production [55]. Palmitic acid (PA) is one of the most abundant FFAs in obesity. In Kupffer cells, the significant accumulation of PA after a high-fat diet causes loss of mitochondrial membrane potential and release of mtDNA [56]. Palmitate also inhibited autophagy and AMP-activated protein kinase (AMPK) activity, resulting in the accumulation of mtROS [57]. Furthermore, palmitate causes mitochondrial fragmentation in macrophage mitochondria by inducing the oligomerization of dynamic-related protein 1 (DRP1). Inhibition of DRP1 reduces palmitate-induced mitochondrial fission [58]. The activation of CD36 by extracellular lipid ligands stimulates nuclear factor-κB (NF-κB) to downregulate ETC components and promote mtROS production and M1-related gene expression [46]. Excessive production of mtROS leads to oxidative stress, resulting in mitochondrial DNA, protein and lipid damage and ultimately mitochondrial dysfunction.

Taken together, hypoxia and FFAs affect mtROS production and mitochondrial dynamics, leading to mitochondrial dysfunction and the release of mtDNA. Hypoxia is also able to shift the cellular energy metabolism from OXPHOS to glycolysis and promote the M1 phenotype.

## 4. Impaired Mitochondrial Quality Control Leads to the Accumulation of Dysfunctional Mitochondria in Macrophages

**Figure 1:**
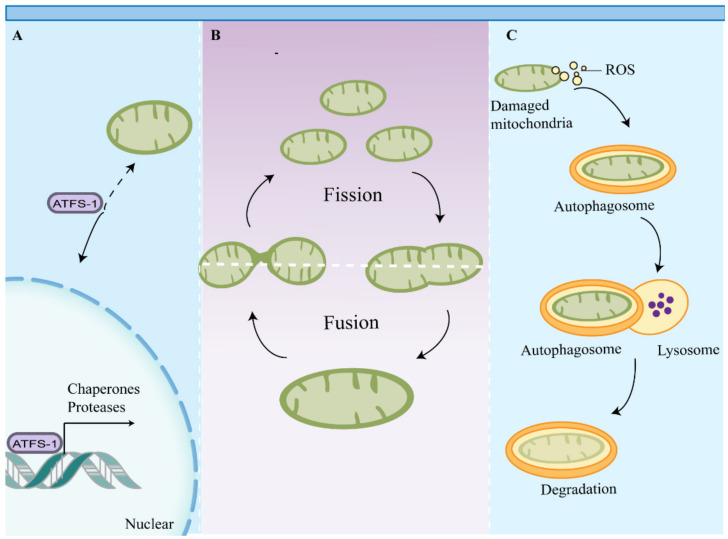
Mitochondrial quality control pathways. Multiple mitochondrial quality control pathways have emerged to maintain mitochondrial function. (**A**) Impaired mitochondrial import induces UPR^mt^, which promotes chaperone and protease gene expression through activation of transcription factors such as ATFS-1. (**B**) Mitochondrial dynamics sustain a balance between mitochondrial fusion and fission to maintain mitochondrial function. (**C**) Mitochondrial components and damaged mitochondria are eventually degraded by mitophagy. Abbreviations: ATFS-1, activating transcription factor associated with stress-1; UPR^mt^, mitochondrial unfolded protein response.

Mitochondria are not only the important energy factory in cells, but also function as intracellular signaling hubs and regulate intrinsic apoptosis and immune responses [59]. The unique properties of mitochondria and mitochondrial metabolism require eukaryotic cells to maintain mitochondrial health and rapidly repair or remove damaged mitochondria to prevent cell death or tissue damage [60]. To this end, multiple MQC pathways have emerged to monitor mitochondrial function and activate appropriate response measures [61] (Figure 1).

MQC includes mitochondrial dynamics, biogenesis, proteostasis, and autophagy, which collectively assist in maintaining the plasticity of metabolic cells [62]. At present, the contribution of mitochondrial fusion and fission to MQC remains unclear, but it is generally believed that there is a sorting of mitochondrial components in this process, with damaged proteins and organelle components separated from the mitochondrial network [63]. Divided mitochondria exhibit different membrane potentials, and mitochondria with higher membrane potentials are more likely to fuse with other mitochondria. Mitochondria with lower membrane potentials are separated from the mitochondrial network and are selectively degraded by mitophagy [64]. Mitochondrial proteins originate from two genomes: mitochondrial and nuclear [65]. Proper mitochondrial function requires synchronization of gene expression in the nucleus and mitochondria and transport of mitochondrial proteins from the cytoplasm to the mitochondria [65]. This highly regulated transport process is dependent on a number of macromolecular protein complexes, including the translocase of the outer membrane of mitochondria (TOM), the translocase of the inner mitochondrial membrane (TIM), and mitochondrial chaperones. Failure to maintain this transport process or disruption of the import machinery leads to mitochondrial uncoupling and induces the mitochondrial unfolded protein response (UPR^mt^) and other mitochondrial quality control pathways, such as mitophagy [66,67]. Mitophagy can selectively remove damaged or dysfunctional mitochondria and is essential for maintaining the quality of the mitochondrial pools. Mitochondrial biogenesis and mitophagy collectively assist in maintaining mitochondrial abundance and efficiency in cells [63].

Adipose tissue of obese and diabetic patients has shown an increased level of autophagy [68], which may be a normal response to the removal of damaged mitochondria. Clinical studies have found more dysfunctional or metabolically impaired mitochondria in obese subjects than in healthy people [69,70]. This suggests that mitophagy may be negatively controlled by excessive fat accumulation or obesity. During overnutrition, activation of mechanistic target of rapamycin complex 1 (mTORC1) inhibits mitophagy, resulting in the accumulation of damaged mitochondria [71]. Lipotoxic ceramide and saturated FA palmitate, which are both elevated in obesity, can activate the NLRP3 inflammasome through autophagy inhibition and accumulation of mtROS [57,72]. Inflammasome activation has been shown to inhibit mitophagy and exacerbate mitochondrial damage [73]. Therefore, inflammasome activation and defective mitophagy may form a vicious cycle, leading to the accumulation of dysfunctional mitochondria and excessive release of pro-inflammatory cytokines.

## 5. Mitochondrial Dysfunction Drives NLRP3 Inflammasome Activation

Accumulation of unfolded proteins, loss of membrane potential, and increased ROS levels induce disruption of mitochondrial membrane integrity, leading to the release of mitochondrial ligands or DAMPs. To date, a variety of mtDAMPs have been identified, including cardiolipin, n-formyl-peptides, mitochondrial transcription factor A (TFAM), mtROS, mtDNA, and the release of locally high ATP. The release of these molecules leads to the activation of inflammasome and the secretion of inflammatory cytokines. Recent studies have shown that mitochondrial dysfunction is associated with NLRP3 inflammasome activation in macrophages.

The inflammasome is a cytoplasmic protein complex that senses both pathogenic and non-pathogenic damage signals. Among them, NLRP3 is the most widely studied inflammasome, which has a unique nucleotide-binding domain (NBD) between the N-terminal pyrin domain (PYD) and the C-terminal leucine-rich repeat (LRR). The NBD is responsible for inflammasome activation and signaling processes, the LRR mediates ligand sensing, and PYD plays an important role in downstream events [18]. The NLRP3 inflammasome consists of NLRP3 itself, apoptosis-associated speck-like protein containing a CARD (ASC), and caspase 1. The ASC has an N-terminal PYD and a C-terminal caspase recruitment domain (CARD). During NLRP3 inflammasome activation, the PYD domain of ASC is interacted with the PYD of NLRP3 through homotypic protein–protein interactions, and the CARD domain binds to the N-terminal CARD domain of caspase-1. In addition to CARD, caspase-1 has a p20 catalytic domain and a C-terminal p10 subunit [74,75,76,77]. After NLRP3 inflammasome assembly, the p10 subunit is cleaved off, and active caspase-1 is generated. The active component containing CARD and p20 subsequently cleaves and activates pro-IL-1β and pro-IL-18 to their active forms [18]. Activated caspase-1 was involved in the regulation of pyroptosis by promoting cleavage of the N-terminal domain of Gasdermin D [76,78].

Mitochondria can directly or indirectly regulate the activation of various inflammasomes. Mitochondria are one of the major sources of intracellular ROS generation. Mitochondrial dysfunction and disruption of the ETC lead to the overproduction of mtROS, which promotes the activation of the NLRP3 inflammasome [79]. Inhibition of OXPHOS and promotion of mtROS production increase NLRP3-dependent caspase-1 activity and promote secretion of IL-1β and IL-18. NLRP3 inflammasome activator can induce the generation of mtROS, and mitochondria-targeted ROS scavengers inhibit ATP- and nigericin-induced production of IL-1β [80]. Mitophagy/autophagy inhibits NLRP3 inflammasome activation by promoting the removal of damaged and dysfunctional mitochondria. However, the role of mtROS in inflammasome activation induced by viral infection is questionable because mitochondria-targeted ROS scavengers do not effectively attenuate IL-1β production following influenza, measles, or encephalomyocarditis virus infection. Furthermore, mtROS plays an important role in inflammasome initiation, but alone is not enough to activate inflammasomes. Therefore, other factors, such as mitochondrial membrane potential, are also important for successful inflammasome activation during viral infection [81]. Under resting conditions, NLRP3 binds to ER and the ASC localizes to mitochondria, cytoplasm and nucleus [79]. Inflammatory body formation requires altered mitochondrial subcellular localization and dynein-mediated migration of mitochondria towards the endoplasmic reticulum, bringing NLRP3 and ASC into close proximity. Inactivation of sirtuin 2 due to impaired mitochondrial homeostasis induces increased α-tubulin acetylation levels, which promote the interaction between ASC and NLRP3 [82]. Calcium signaling also activates the NLRP3 inflammasome by promoting mitochondrial damage. In response to ATP stimulation, mitochondrial damage induced by calcium mobilization leads to the release of mtDNA and the generation of mtROS. Multiple NLRP3 inflammasome activator can induce calcium mobilization, promoting mitochondrial damage due to calcium overload [83,84]. The bacteria-like CpG islands allow mtDNA to be recognized by the innate immune system and activate the immune response. Nakahira, who first linked mtDNA to NLRP3 inflammasome activation, found that LPS can induce the accumulation of mtDNA in the cytoplasm of macrophages, thereby promoting caspase-1 activation and IL-1β/IL-18 secretion. In this process, mtROS promotes the release of mtDNA into the cytoplasm, which in turn activates the NLRP3 inflammasome [85]. NLRP3 also appears to bind oxidized mtDNA and stabilize it in the cytoplasm [86]. Cardiolipin is required for the normal function of mitochondria. Mitochondrial dysfunction induces the release of cardiolipin, which exhibits the properties of DAMPs [87]. Knockdown of cardiolipin synthase alleviates NLRP3 inflammasome activation by silica and ATP. MtROS can also promote activation of the NLRP3 inflammasome by oxidizing other mtDAMPs, such as mtDNA and cardiolipin, increasing their stimulatory capacity or further damaging mitochondria and other organelles [88].

## 6. Mitochondrial Dysfunction Impairs Macrophage Function and Plasticity

Mitochondria are one of the major targets of cellular stress caused by inflammation, pathogen infection and aging. In inflammatory macrophages, mitochondrial stress promotes the release of mtDAMPs, which stimulate innate immune receptors and downstream pathways, suggesting that mitochondria are both targets and instigators of inflammatory responses [89]. Accumulation of mitochondrial damage and the reduction in the efficiency of energy production reduce the capacity of the immune system to respond to viral infection by reducing type I interferon (IFN-I) release [90]. Mitochondria are one of the major sources of ROS in M1 macrophages. MtROS can not only induce the activation of pro-inflammatory pathways and promote the release of inflammatory cytokines, but also promote the anti-bactericidal effect of macrophages. In addition to microorganisms, efficient phagocytosis by macrophages is critical for the clearance of aged or damaged cells and maintenance of tissue homeostasis [91,92]. Diminished macrophage phagocytosis not only makes us vulnerable to infection, but also leads to tissue damage and the accumulation of apoptotic and senescent cells in the body, leading to the continuous activation of immune cells and more oxidation and inflammation [93].

In response to different environmental conditions and stimuli, changes to mitochondrial metabolism and physiology allow macrophages to polarize to pro-inflammatory M1 phenotype or anti-inflammatory M2 phenotype. Altered macrophage metabolism leads to changes in macrophage function/phenotype, and vice versa. The plasticity of metabolism enables macrophages to respond rapidly to changes in environmental conditions. Mitochondria, as the centers of cellular energy metabolism, play an important role in the metabolic reprogramming. Mitochondrial dysfunction leads to impairment or loss of cellular metabolic plasticity in macrophages, which are then not able to respond quickly to specific stimuli, leading to macrophage dysfunction and the development of certain diseases. Mitochondrial dysfunction induces complex energy metabolism reprogramming, including decreased mitochondrial oxidative metabolism, increased glucose uptake, and enhanced glycolysis [94]. Enhanced glycolysis will promote the synthesis of NADPH, which induces the generation of ROS and NO by activating iNOS and NOX. NO reacts with superoxide to generate peroxynitrite, which leads to the accumulation of ROS [95]. NO inhibits mitochondrial OXPHOS while ROS stabilizes HIF-1α, which further promotes glycolysis [96]. Inhibition of OXPHOS prevents the repolarization of M1 macrophages to M2 macrophages. Restoring mitochondrial function may be useful to improve the reprogramming of inflammatory macrophages into anti-inflammatory cells [25].

In summary, mitochondria play an important role in the regulation of macrophage function. On the one hand, mitochondrial dysfunction leads to changes in cellular metabolism and the release of DAMPs, which drives the activation of inflammatory macrophages. On the other hand, mitochondria also effect macrophage phagocytosis by ROS generation.

## 7. Adipose Tissue Macrophages Link Adipose Tissue Inflammation to Insulin Resistance

**Figure 2:**
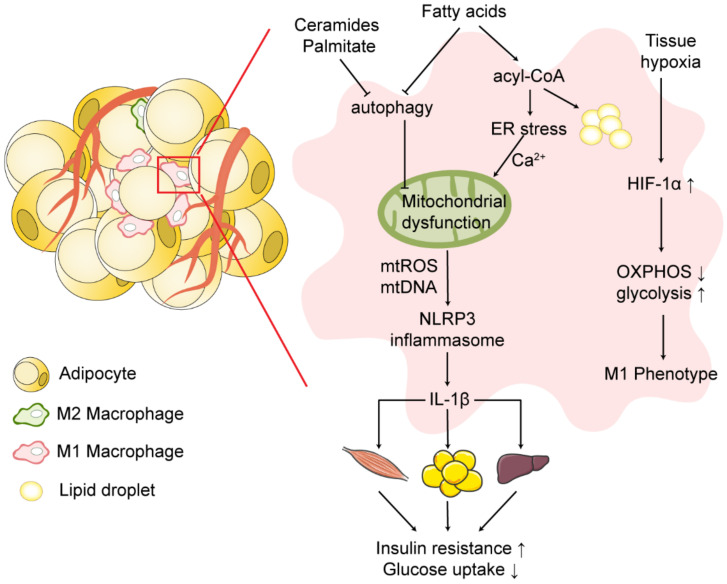
Mitochondrial dysfunction and NLRP3 inflammasome activation in obese adipose tissue. During obesity, the hypertrophy and hyperplasia of adipose tissue induce local tissue hypoxia and the infiltration of macrophages in adipose tissue. Under hypoxia, activated HIF-1α shifts cellular energy metabolism from OXPHOS to glycolysis and induces the macrophage M1 phenotype. Excessive uptake of free fatty acids by adipose tissue macrophages shifts fatty acid utilization from OXPHOS to triglyceride, phospholipid, and ceramide synthesis, promoting lipotoxicity in macrophages. Fatty acids induce disruption of ER structure and ER stress, leading to mitochondrial calcium overload and mitochondrial dysfunction. Impaired autophagy induced by ceramides, palmitates and saturated fatty acids leads to the accumulation of dysfunctional mitochondria. Mitochondrial dysfunction induces activation of the NLRP3 inflammasome and release of IL-1β, promoting the progression of insulin resistance. Symbols: ↑ = increase; ↓ = decrease. Abbreviations: OXPHOS, oxidative phosphorylation; ER, endoplasmic reticulum; HIF-1α, hypoxia-inducible factor-1α.

Adipose tissue is not only an important organ that store excess energy in the form of fatty molecules, but also a key site for inflammatory responses and mediators. During obesity, the altered adipose tissue microenvironment induces macrophage polarization to a pro-inflammatory M1 phenotype and release of inflammatory cytokines, impairing insulin signaling and promoting the progression of insulin resistance (Figure 2).

Chronic inflammation in adipose tissue was identified as a key risk factor for insulin resistance and T2D in obese individuals [97]. In general, obese individuals with insulin resistance exhibit high levels of adipose tissue inflammation, whereas adipose tissue in insulin-sensitive obese individuals exhibits limited inflammatory properties [98,99]. Macrophages are the most abundant innate immune cells in obese adipose tissue, accounting for 50% of the total number of cells. Besides macrophages, there are other types of immune cells involved in adipose tissue inflammation during obesity. Although certain T and B cell subsets play important roles in regulating adipose tissue inflammation, macrophages are widely believed to be the primary effector cells responsible for diminished insulin signaling [100,101]. Besides increasing in numbers, macrophages also exhibit an altered phenotype during obesity. In lean adipose tissue, macrophages mainly exhibit an M2 phenotype at steady state. In contrast, ATMs in obese patients polarize to a pro-inflammatory M1 phenotype [15,102,103]. M1 macrophages secrete multiple pro-inflammatory cytokines, such as TNF-α and IL-1β. IL-1β has been considered as a key factor in the pathogenesis of T2D. IL-1β promotes the activation of the IKK/NF-κB pathway. In adipose tissue in obesity, the activation of this pathway results in insulin resistance through IKK-mediated serine phosphorylation of IRS-1 or insulin receptor, leading to the inhibition of insulin-induced serine phosphorylation and downstream signaling [104]. IL-1β can also activate c-Jun NH(2)-terminal kinases (JNKs) and other MAPKs, which induces insulin resistance by mediating the serine and threonine phosphorylation of IRSs and impairing the interaction between IRS and insulin receptor and downstream insulin signaling [104]. Besides directly inhibiting insulin signaling by reducing the phosphorylation of ISR-1 or inhibiting its expression, IL-1β promotes the production of other inflammatory cytokines [105], such as TNFα and IL-1β itself, by activating the NF-κB pathway, which initiates a self-amplifying cytokine network [106]. These cytokines can lead to decreased insulin sensitivity of insulin target cells (adipocytes, hepatocytes, and muscle cells) through paracrine signaling or infiltration into the systemic circulation, promoting the progression of insulin resistance and systemic hyperglycemia [107]. Unlike adipocytes, macrophages are insensitive to insulin and retain the ability to take up glucose upon insulin resistance [108]. Glucose uptake by macrophages appears to be dependent only on the availability of glucose, and glucose directly enters into the glycolysis pathway and promotes the M1 phenotype [108,109]. A recent study showed that ATMs in obese mice secrete exosomes that target muscle and liver cells. Injection of exosomes derived from ATMs of obese mice into lean mice leads to increased glucose tolerance, insulin resistance, and glucose-stimulated insulin secretion, which may be mediated by the transfer of microRNAs that regulate insulin sensitivity [110,111].

Adipose tissue stored in various locations throughout the body has specific metabolic and inflammatory properties. Based on anatomical location, adipose tissue can be divided into subcutaneous fat and visceral fat, which have different contributions to the development of metabolic abnormalities. Epidemiological evidence suggests that visceral fat mass is the major risk factor for the development of metabolic abnormalities, including insulin resistance [112]. Metabolically healthy obese individuals, characterized by more subcutaneous fat, less visceral fat and ectopic liver fat, do not exhibit the metabolic disturbances associated with obesity and are at lower risk for T2D and cardiovascular disease [113]. Studies have shown that visceral adipocyte mass is negatively correlated with insulin sensitivity. Hypertrophic adipocytes lead to ER and mitochondrial stress that triggers adipocyte death and adipose tissue inflammation. In vitro, visceral fat releases more inflammatory cytokines than subcutaneous fat [114]. Some studies have found that visceral fat has more macrophages [115,116], but other studies suggest that there is no significant difference in the number of macrophages between visceral fat and subcutaneous fat [114]. Adipose tissue microenvironment plays key roles in the macrophage phenotype. The differences in macrophage phenotype caused by the different microenvironments of these two adipose tissues may be the reason why visceral fat releases more inflammatory cytokines [116,117,118].

## 8. Mitochondrially Targeted Treatment Strategies

### 8.1. Lifestyle

Mitochondrial dysfunction contributes to the progression and severity of a wide range of diseases, including diabetes, cardiovascular disease and neurodegenerative diseases. Physical exercise reduces the risk and slows the progression of diabetes because it improves glycemic control [119] and insulin sensitivity in obese patients [120]. Exercise works primarily by acting on mitochondria. Exercise training can restore mitochondrial function and insulin sensitivity in T2D subjects [121]. In contrast to chronic elevation of ROS production caused by mitochondrial dysfunction and overnutrition, which has pathological effects leading to insulin resistance and diabetes, transient and low-intensity ROS production caused by physical activity is a signal that promotes beneficial effects, such as mitochondrial biogenesis and mitophagy [122,123,124]. Recent studies have shown that physical exercise can improve insulin sensitivity and mitochondrial biogenesis in skeletal muscle of patients with T2D. Muscle mitochondrial respiration and mitochondrial content, oxidase activity, and mitochondrial density are significantly increased in T2D patients after exercise [125]. Exercise can also activate AMPK, leading to the activation of PGC-1α by increasing threonine and serine phosphorylation, which promotes mitochondrial biogenesis [126]. Furthermore, exercise training can activate sirtuin1 (SIRT1) by elevated NAD+/NADH ratio and leads to deacetylation-dependent PGC-1α activation [127]. Regular exercise can also trigger multiple signaling pathways involved in skeletal muscle mitochondrial biogenesis, dynamics and metabolism [128]. Conversely, reduced physical activity and sedentary lifestyles are a major contributor to the rise in obesity, T2D, and many other metabolic disorders [129].

In addition to regular exercise, calorie restriction (CR) is a promising non-genetic and non-pharmaceutical nutritional intervention that can increase longevity and prevent metabolic disorders [130,131]. At present, the molecular mechanisms by which CR produces therapeutic effects in metabolic-related diseases remains unclear. It has been found that CR can enhance mitochondrial function by reducing ROS production and oxidative damage [132]. Increased AMPK expression has been observed in different experimental models of CR, which promotes the expression of several genes involved in mitochondrial biogenesis and adaptation by phosphorylation of PGC-1α [133,134,135]. NADH and NAD+ are important regulators of many enzymes and transcription factors. CR-induced increases in NAD+ can promote sirtuin-dependent activation of PGC-1α [136]. CR can also induce mitochondrial biogenesis and improve mitochondrial function via PGC-1α-dependent mechanisms [136]. Moreover, CR is associated with a more connected mitochondrial network and increased mitophagy, both of which are required to maintain a healthy mitochondrial network [136]. CR also significantly improves insulin sensitivity in many species, including humans, non-human primates, mice, and rats, so CR may be an effective treatment for obesity and insulin resistance [137,138,139]. Data from a variety of epidemiological, clinical, and experimental studies suggest that CR has beneficial effects on health via inhibiting key nutrient sensing and inflammatory pathways and is a cornerstone for the prevention and treatment of metabolic disorders [140]. CR also has some limitations. Studies have shown that long-term CR increases the risk of developing several diseases such as osteoporosis, slow wound healing, and sensitivity to cold environments [141].

### 8.2. Pharmacological Therapy

Many drugs used to treat diabetes and other metabolic disorders have recently been shown to affect mitochondrial function, which is to be expected because they target energy metabolism in some way (Table 1). Thiazolidinediones (TZDs) are antidiabetic drugs that targets PPAR-γ, promoting the storage of FA and inhibiting its export. [142]. Activation of PPAR-γ can also induce the macrophage M2 phenotype and improve insulin resistance [143]. In addition to improving insulin resistance by activating the PPAR family of nuclear receptors, TZDs can also promote shifts in cell metabolism by directly inhibiting complex I. Metformin is one of the most widely used antidiabetic drugs. Recent studies have shown that metformin is not only able to improve chronic inflammation by improving metabolic parameters, but also has a direct anti-inflammatory effect [144]. Metformin can maintain mitochondrial integrity by inhibiting dynamin-related protein 1 (DRP 1), which reduces NLRP3 inflammasome activation and adipose tissue inflammation [145]. Metformin can also inhibit endotoxin-induced production of pro-IL-1β in bone marrow-derived macrophages by inhibiting mitochondrial complex I and ROS production [146].

Oxidative stress caused by overproduction of mtROS appears to be a major risk for the development of metabolic diseases and chronic inflammation. Therefore, drugs that reduce intracellular ROS overproduction may be potential therapeutic options to improve mitochondrial function in these diseases. Clinical studies have shown that mitochondrial antioxidants, such as vitamin E, N-acetylcysteine, glutathione, and coenzyme Q10, can reduce mtROS production and improve hyperglycemia in diabetic patients [147]. In addition, other mitochondrial antioxidants such as ubiquinone (MitoQ) and vitamin E (MitoVitE) have been used to overcome mitochondrial dysfunction [148]. MitoQ is a mitochondria-specific antioxidant, and its lipophilic triphenylphosphine (TPP) cation can bind to the antioxidant site of ubiquinone [149]. Mitochondria-selective accumulation of MitoQ mediated by lipophilic TPP cation reduces mtROS and improves mitochondrial function [150]. Recent studies have shown that MitoQ can suppress NLRP3 inflammasome by scavenging intracellular and mitochondrial ROS [151,152]. Mito-TEMPO, a mitochondria-targeted superoxide dismutase mimetic, can also suppress NLRP3 inflammasome [153] and improve insulin resistance and metabolic dysfunction [154].

The mitochondrial quality control system is essential for the maintenance of mitochondrial function and the clearance of damaged mitochondria. Impaired mitochondrial quality control plays an important role in the development of diabetes and diabetes complications. Strategies targeting mitochondrial quality control mechanisms may be promising therapies for the treatment of diabetes and diabetes complications. Mitochondrial fission leads to ROS overproduction, and mitochondrial fission inhibitors, such as mdivi 1, P110 and dynasore, can effectively improve mitochondrial stress [155,156,157]. Inhibition of mitochondrial fission plays a protective role against diabetes. Metformin and resveratrol maintain mitochondria integrity by inhibiting DRP1, preserving cell function under hyperglycemic conditions [157]. Empagliflozin significantly reduces the expression of phosphoglycerate mutase family member 5 (PGAM5) by activating AMPK and inhibits the fission of mitochondria, improving diabetic renal tubular injury [158]. Facilitating the clearance of damaged and dysfunctional mitochondria by enhancing mitophagy is also beneficial for the treatment of diabetes. MitoQ reverses the deficient mitophagy by up-regulating PINK1 and Parkin expression and inhibiting mtROS generation in diabetic nephropathy [159]. Recombinant human progranulin (RPGRN) can alleviate high glucose-induced mitochondrial dysfunction by promoting mitophagy and mitochondrial biogenesis [160].

**Table 1:** The effects of antidiabetic drugs on mitochondrial function.

Substance	Pathway or Category	Effects on Mitochondria	References
TZDs	PPARs agonistic	Enhanced fatty acid oxidation	[161]
Metformin	Complex I inhibition	Inhibited respiratory chain	[162]
Pioglitazone	PPAR-γ/PGC-1α Pathway	Improved mitochondrial biogenesis and dynamics	[163]
Resveratrol	SIRT1-PGC-1α axis	Induced mitochondrial biogenesis	[164]
MitoQ	Nrf2/PINK1 pathway	Restored mitophagy	[159]
Mdivi1	Inhibitor of Drp1	Inhibited mitochondrial fission	[165]
Dynasore	Inhibitor of Drp1	Inhibited mitochondrial fission	[166]
Empagliflozin	AMPK/SP1/PGAM5 pathway	Inhibited mitochondrial fission	[167]
rPGRN	PGRN- SIRT1-PGC-1α/FoxO1 pathway	Enhanced mitochondrial biogenesis and mitophagy	[160]

Abbreviations: TZDs, thiazolidinediones; PPAR, peroxisome proliferator-activated receptor; PGC-1α, peroxisome proliferator-activated receptor-gamma co-activator-1alpha; SIRT1, sirtuin1; Nrf2, nuclear factor erythrocyte 2-related factor 2; PINK1, PTEN-induced kinase 1; Drp1, dynamin-related protein 1; AMPK, AMP-activated protein kinase; SP1, specificity protein 1; PGAM5, phosphoglycerate mutase family member 5; rPGRN, recombinant human progranulin; FoxO1, forkhead box O1.

### 8.3. Novel Mitochondria-Targeted Therapeutic Approaches

Given the important role of mitochondrial function in macrophage polarization, strategies that can restore mitochondrial function may reduce adipose tissue inflammation and insulin resistance by regulating macrophage metabolism. Studies have shown that a variety of natural compounds can restore mitochondrial function and prevent mitochondrial-related diseases. Therefore, the development of mitochondrial modulators from natural compounds may be a potential strategy for the treatment of mitochondrial-related diseases. Various dietary natural compounds, such as polyunsaturated fatty acids and polyphenols, can prevent or even treat metabolic diseases by restoring mitochondrial function [168]. Multiple dietary polyunsaturated fatty acids can prevent the development of systemic insulin resistance. Intake of α-linolenic acid increases the expression of PGC-1α and exerts anti-obesity effects by promoting mitochondrial biogenesis and β-oxidation [169]. The dietary polyphenol resveratrol mainly exists in grape skins and red wine and exerts antioxidant function by reducing ROS production. Resveratrol activates PGC-1α in a SIRT1-dependent manner and increases systemic insulin sensitivity, mitochondrial biogenesis, and oxidative capacity [170]. In addition, other polyphenols, such as quercetin and hydroxytyrosol, can also promote mitochondrial biogenesis by activating the SIRT1-PGC-1α pathway [171].

Recent studies have shown that various herbs or their extracts can regulate mitochondrial function. Ginsenoside Rg3, one of the main components in ginseng, can promote mitochondrial biogenesis and antioxidant capacity in Sprague–Dawley rat cardiomyocytes [172]. Korean red ginseng preserves mitochondrial function and protects against intracellular inflammation in diabetic mice [173]. Curcumin, a phenolic compound derived from turmeric, restores mitochondrial function in liver and kidney tissues of diabetic db/db mice [174]. In addition, various herbal extracts such as berberine, salidroside, shikonin and syringic acid can also restore mitochondrial function and improve diabetes and its complications through different mechanisms [175,176,177,178]. Although researchers have carried out extensive research on the mechanisms of traditional Chinese medicine (TCM), the effects of TCM on mitochondria and its therapeutic effect on metabolic diseases remains unclear, which limits the application of TCM. It is undeniable that TCM or integrated traditional Chinese and Western medicine may be an effective approach to treat diabetes and its complications.

Besides these mitochondria-targeted drugs or compounds, mitochondria-targeted gene therapy and mitochondrial transplantation can also restore mitochondrial function in diabetic patients. Gene therapy restores mitochondrial function in T2D by correcting mitochondrial omics [179]. Mitochondrial transplantation aims to transfer functional exogenous mitochondria into mitochondria-defective cells to restore mitochondrial function [180]. Although mitochondrial transplantation has been shown to be a promising approach to treat mitochondrial-related diseases, several challenges remain to be addressed, such as mitochondrial isolation and preservation methods, mitochondrial delivery methods, and enigmatic mechanisms orchestrating mitochondrial incorporation. Implantation of mitochondria into unwanted tissue may lead to side effects [180].

## 9. Conclusions and Remarks

The global obesity epidemic has led to increased risk of T2D. During obesity, an increased inflammatory response has been found in multiple tissues, including adipose tissue and liver. Adipose tissue inflammation seems to play a unique role in the pathology of obesity. Inflammation in liver is resolved after weight loss, while adipose tissue remains inflamed. In humans, this response is highly variable. After extreme weight loss, adipose tissue inflammation is resolved in some individuals with obesity, but not in others [181]. The effects of this obesogenic memory in adipose tissue on obesity-associated morbidity and mortality remains unclear. Studies have found that being overweight or obese during one’s lifetime increases mortality, regardless of subsequent weight loss [182]. Although inflammation in other tissues during obesity also contributes to the development of insulin resistance, low-grade adipose tissue inflammation during obesity is considered as an important mechanism leading to insulin resistance. Inflammatory changes in human adipose tissue during obesity are less pronounced than changes in mice, although the presence of CLSs was positively associated with a worsening level of insulin resistance. In addition, there may be interindividual differences in the degree of inflammation in adipose tissues in humans, and the importance of macrophage-mediated adipose tissue inflammation in the development of insulin resistance may be different among obese individuals [183].

The cause of adipose tissue inflammation remains unclear. We believe that macrophage mitochondrial dysfunction in obese adipose tissue plays an important role in inducing the inflammatory response of macrophages and the release of inflammatory cytokines. During obesity, local hypoxia and increased FFAs caused by adipose tissue expansion induce mitochondrial dysfunction and altered metabolism in adipose tissue macrophages, eventually leading to macrophage M1 polarization. The activation of inflammasome links obesity, insulin resistance and development of T2D. Activation of the NLRP3 inflammasome induces the release of IL-1β, leading to a decreased insulin sensitivity in insulin-targeted cells. The NLRP3 inflammasome and its products IL-1β and IL-18 may be potential targets for diabetes therapy. Currently, several specific inhibitors of these ILs are being widely studied as promising therapeutics for many diseases, including T2D. The activation of the NLRP3 inflammasome is regulated by a wide variety of factors and conditions. In this review, we focused on the role of mitochondrial dysfunction in NLRP3 inflammasome assembly and activation, but many key questions about the mechanism need to be addressed. The elucidation of the regulatory mechanism between mitochondrial dysfunction and NLRP3 inflammasome activation will facilitate the exploration of new therapeutic targets.

Inflammation in obese patients provides new perspectives for the therapeutic strategies for T2D and other metabolic diseases. Further research in this area could facilitate the development of new treatments or the repurposing of existing drugs, for example, using anti-inflammatory drugs for metabolic diseases and using metabolic therapies for inflammatory diseases. Anti-inflammatory strategies have promising effects in improving insulin resistance in obesity mouse models. However, targeting only one inflammatory cytokine may not be sufficient, and the possible side effects of immunosuppression should be considered when using anti-inflammatory agents or inflammasome inhibitors. Therefore, better therapies are needed to prevent obesity-related inflammation from increasing insulin resistance and T2D. Mitochondrial dysfunction leads to macrophage M1 polarization and the massive release of inflammatory cytokines. The clearance of dysfunctional mitochondria by autophagy/mitophagy and the restoration of mitochondrial biogenesis contribute to the resolution of inflammation. This suggests that restoring the homeostasis of the macrophage mitochondrial network may be a good strategy for diabetes prevention and treatment. Many antidiabetic drugs have been shown to affect mitochondria and macrophage polarization, including metformin, TZDs and some mitochondria-targeted antioxidants. Therefore, expanding our understanding of adipose tissue macrophage polarization induced by mitochondrial dysfunction may shift therapeutic targets from inflammatory cytokines to mitochondrial function in ATMs. Therapeutic modalities that can restore mitochondrial network homeostasis may be used to reprogram macrophage metabolism, which enables macrophages to respond to metabolic challenges and maintains adipose tissue homeostasis during obesity.

## Data Availability

Not applicable.
